# An Unusual Case of Metastatatic Renal Cell Carcinoma Presenting as Melena and Duodenal Ulcer, 16 Years After Nephrectomy; a Case Report and Review of the Literature

**Published:** 2015-03

**Authors:** Bita Geramizadeh, AmirAhmad Mostaghni, Zeinab Ranjbar, Farid Moradian, Mina Heidari, Mohammad Bagher Khosravi, Seyed Ali Malekhosseini

**Affiliations:** 1 Shiraz Transplant Research Center, Nemazee Hospital, Shiraz University of Medical Sciences, Shiraz, Iran;; 2 Department of Pathology, School of Medicine, Shiraz University of Medical Sciences, Shiraz, Iran;; 3 Department of Internal Medicine, School of Medicine, Shiraz University of Medical Sciences, Shiraz, Iran;; 4 Department of Surgery, Shahid Faghihi Hospital, Shiraz University of Medical Sciences, Shiraz, Iran;; 5 Department of Anesthesiology, School of Medicine, Shiraz University of Medical Sciences, Shiraz, Iran

**Keywords:** Duodenal ulcer, Gastrointestinal hemorrhage, Renal cell carcinoma, Pancreaticoduodenectomy

## Abstract

Renal cell carcinoma comprises about 2% of adult tumors. The overall 10-year survival rate of patients with RCC after nephrectomy is about 18-27%. The incidence of metastasis of initial RCC is about 24-28%, but this rate after nephrectomy is as high as 51%. The most common site of recurrence is the lung, however liver and bone metastases are common.

There are many reported cases with late metastasis, however isolated late metastasis in the gastrointestinal tract especially duodenum is very rare.

Herein we report our experience with a case of gastrointestinal bleeding secondary to metastatic renal cell carcinoma to duodenum, 16 years after nephrectomy.

To the best of our knowledge, about 30 of such cases have been reported in the English literature. Many of the previous cases have been part of disseminated disease and isolated duodenal metastasis is very rare. The longest reported duration between nephrectomy for renal cell carcinoma and duodenal metastasis has been 13 years, thus it seems our case to be also unique because of very late duodenal metastasis.

## Introduction

Renal cell carcinoma (RCC) as the most common malignant tumor of the kidney has an unpredictable and bizarre natural history, i.e. on the one hand it has an indolent growth rate and showed metastasis at the time of presentation and on the other hand it can remain stable years after nephrectomy.^1^

The most common sites of metastasis in RCC are the lung and bone; other less common metastatic locations are lymph nodes, adrenal, liver, opposite kidney and brain.^2^

Gastrointestinal metastasis of RCC is rare and reported in 4% of the cases, however, in the GI tract, the least common site of metastasis is small intestine and it is extremely rare for metastatic RCC to be presented as duodenal ulcer and melena.^3^

To the best of our knowledge, less than 30 cases of duodenal metastasis of RCC have been reported in the English literature and the longest duration after nephrectomy has been 13 years. 

Herein we report our experience with a 61-year-old man presented with melena 16 years after nephrectomy that was diagnosed to be metastatic RCC. The patient underwent Whipple’s operation with excellent postoperative course. 

## Case Report

A 61-year-old man was presented with melena. He was in a good health condition. His past medical history showed right kidney nephrectomy (16 years ago) with the diagnosis of clear cell RCC without any further treatment. He had been completely normal during the last 16 years.

Laboratory findings were:

- Liver function tests were normal: ALT=19 U/L (normal<40), AST=25 U/L (normal<40), Alkaline phosphatase=193 U/L (normal 80-306)
- Complete Blood Count was normal: WBC: 6000/µl, RBC: 5×10^6^/µl, and Platelet: 263000/µl

Physical examination showed normal heart and lung examination. There was no lymphadenopathy. Blood pressure, pulse rate, heart rate, and temperature were all unremarkable.

**Figure 1:**
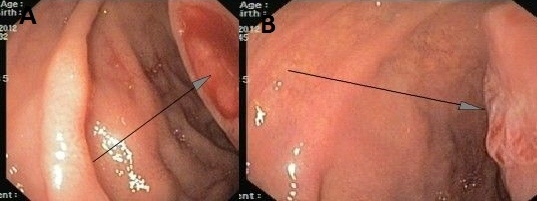
(A and B) Endoscopy of the duodenum shows ulcers (Arrows).

**Figure 2:**
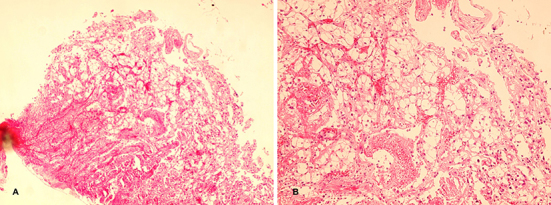
High and low power view of duodenal ulcer biopsy show collections of clear cells. (A: H&E ×100, B: H&E ×250).

**Figure 3:**
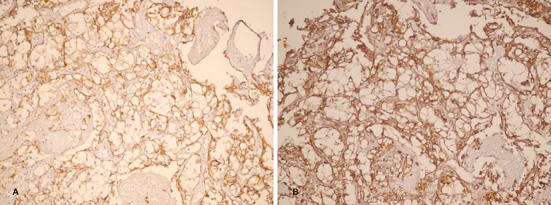
Immunohistochemistry was positive for cytokeratin (A) and vimentin (B).

Stool occult blood test was positive in several occasions, so the patient underwent upper and lower gastrointestinal endoscopy. An ulcer was found in the second part of duodenum with fine oozing of blood (figure 1a, 1b as indicated by arrows). A biopsy was taken and sections from duodenum showed surface ulceration beneath of which there was subepithelial collection of cells with clear cytoplasm (figure 2a, 2b). Immunohistochemistry was positive for cytokeratin, CD10, vimentin, and RCC antibody (figure 3a, 3b).

**Figure 4:**
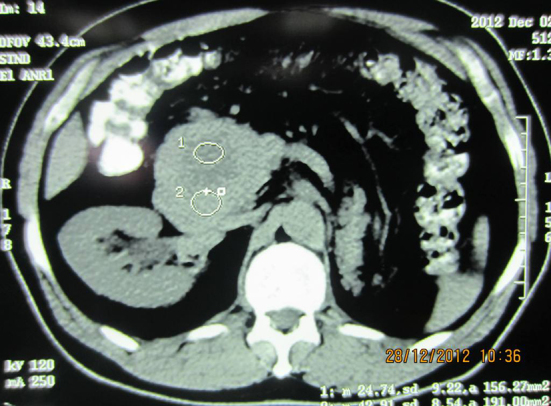
Abdominal CT scan shows pancreaticoduodenal mass and absence of right kidney.

**Figure 5:**
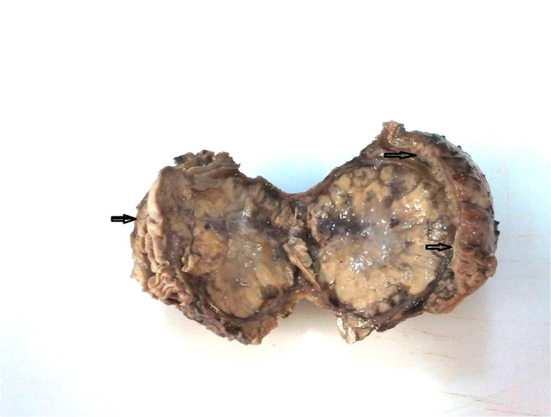
Gross specimen of the duodenal mass shows relatively well defined mass beneath the small intestinal wall (arrows show mucosa of duodenum).

**Figure 6:**
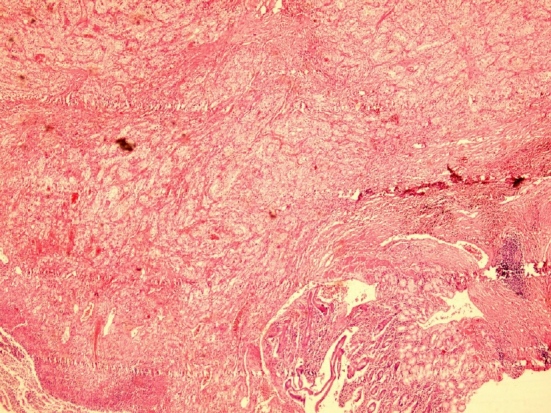
Sections from ampulla of vater show subepithelial tumoral tissue with clear cell morphology. (H&E ×100)

With the pathologic diagnosis of metastatic RCC, abdominal CT scan was performed that showed a mass in the distal pancreas measuring 7 cm in greatest diameter with central necrosis, most probably arisen from duodenum extending to the pancreas (figure 4). Whipple’s operation was performed and the pancreatoduodenal mass was resected. The specimen showed a mass measuring 7×5.5 cm in the duodenum extending to the head of the pancreas (figure 5). Pathology of the specimen showed metastatic RCC involving the duodenum and distal pancreas (figure 6). The patient was discharged in good condition to be followed for further evaluation.

This case is being reported by the informed consent of the patient. 

## Discussion

The behavior of RCC is completely unpredictable. Metastatic masses can be the first presentation of RCC or can be diagnosed years after the initial diagnosis and nephrectomy.^4^ Most common sites of metastatic RCC are the liver and lung.^2^ Metastases to the pancreas and small intestine are rare, but can present as gastrointestinal bleeding*.*^5^ About 4% of RCCs metastasize to the small intestine, of these; the duodenum is the least frequent site and can be involved by direct invasion of the tumor, or through lymphatic, transcoelomic or haematogenous spread and also direct invasion of pancreatic metastasis.^5^ In our patient, at the time of surgery, the main bulk of tumor was in the duodenum with extension to the head of pancreas.

According to the previous reports, duodenal involvement of RCC can be presented with jaundice, anemia and gastrointestinal bleeding, malabsorption and obstruction.^6^ Primary presentation of metastatic RCC as gastrointestinal bleeding has been rarely reported.^7^

The longest duration between nephrectomy and duodenal metastasis was 13 years in previous reports.^8^

Diagnosis of duodenal metastasis in RCC most commonly has been made by endoscopy. On endoscopy, the lesion can be seen as an ulcer, submucosal mass with ulceration or multiple nodules or small polyps of varying sizes.^9^

Some patients present with concomitant metastasis in more body locations or other segments of the intestine such as the colon and duodenum.^10^

Our case presented with melena and duodenal ulcer 16 years after right nephrectomy for RCC. In previous studies, most of the duodenal metastasis of RCC was after right kidney nephrectomy.^8^

Most of the metastatic RCCs in the duodenum were primarily in the second portion with and without pancreatic involvement.^9^

The treatment of choice for localized metastatic RCC is surgery.^2^ In previous reports, most of the patients with duodenal metastasis of RCC were treated with Whipple’s operation; however, there were successful surgeries of duodenal saving segmental or wedge resection.^3^^,^^11^ Any type of metastasectomies can increase the survival of the patient.^5^

To the best of our knowledge, less than 30 cases of RCC with duodenal metastasis have been reported in the English literature (table 1). The longest duration after nephrectomy has been 13 years. Our case presented with melena 16 years after right nephrectomy for RCC, which to the best of our knowledge is the longest duration after nephrectomy.

**Table 1:** Clinical characteristic of the previous cases of duodenal metastasis of RCC

**Author/Year**	**Sex/Age**	**Presenting symptom**	**Concomitant other organ involvement**	**Years after initial nephrectomy **	**Treatment**
Lawson et al./1966^12^	69/F	Anemia , GI bleeding	-	0	Whipple’s operation
Heyman et al./1978^13^	64/M	GI bleeding	Colon	8 years	Complex resection
McNicholas/1981^14^	52/M	Malabsorption	-	10 years	No surgery
Lynch et al./1987^15^	67/M	GI bleeding	Pancreas	2 years	No surgery
Lynch et al./1987^15^	61/M	Jaundice	-	6 years	No surgery
Lynch et al./1987^15^	16/M	GI bleeding	-	1 year	No surgery
Robertson et al./1990^16^	70/M	GI bleeding	Pancreas	13 years	Whipple’s operation
Toh et al./1996^2^	59/F	Anemia, Bowel obstruction	-	10 years	Segmental resection
Ohmura et al./2000^17^	62/M	Obstruction	-	5 years	-
Hashimoto et al./2000^18^	57/M	GI bleeding	Pancreas	11 years	Pancreaticoduodenectomy
Nabi et al et al./2001^19^	40/M	Obstruction	-	4 years	Segmental resection
Lee et al./2002^10^	76/F	Abdominal pain	Colon	4 years	No surgery
Loualidi et al./2004^6^	76/M	Anemia, GI bleeding	-	5 years	No surgery
Chang et al./2004^20^	63/F	GI bleeding	-	9 years	Segmental resection
Bhatia et al./2006^9^	50/M	Jaundice	Liver	1 year	No surgery
Sadler et al./2007^5^	67/M	GI bleeding	-	0	No surgery
Sadler et al./2007^5^	75/M	Anemia	-	9	No surgery
Adamo et al./2008^21^	86/F	Anemia, Obstruction	-	9 years	Pancreaticoduodenectomy
Eo et al./2009^22^	47/M	Intussception	Lung	2 years	Segmental resection
Teo MY/2010^8^	50/F	Jaundice	Lung	1 year	No surgery
Rustagi et al./2011^23^	66/M	GI bleeding	-	13 years	Whipple’s operation
Cherian et al./2011^24^	80/M	GI bleeding	Lung and Bone	11 months	No surgery
Vashi et al./2011^25^	53/M	GI bleeding	-	2 weeks	Segmental resection
Chua et al./2011^26^	56/M	GI bleeding, Anemia	Lung	0	Segmental resection
Zhao et al./ 2012^3^	56/M	GI bleeding	-		Whipple’s operation
Yang et al./2012^4^	72/M	GI bleeding	-	10 years	Whipple’s operation
Ashraf Teli et al./2012^7^	52/M	GI bleeding	Liver	8 years	Segmental resection
Current Case	61/M	GI bleeding	-	16 years	Whipple’s operation

As a conclusion, distant metastasis of RCC can be presented very late with unusual and unpredictable symptoms. In all patients with a history of RCC, GI bleeding should be considered as a possible cause of metastasis. 
